# Unlocking the promise of virtual care in hospitals: the Smarter Hospitals Project protocol

**DOI:** 10.1186/s12913-025-13129-2

**Published:** 2025-08-11

**Authors:** Reema Harrison, Rebecca Mitchell, Ramya Walsan, Maryam Sina, Robyn Clay-Williams, Alexander Cardenas, Michelle Moscova, Dalal Baumgartner, Mashreka Sarwar, Johanna Westbrook, Elizabeth Manias, Natalie Taylor, Rebecca Lawton, Sabe Sabesan, Virginia Mumford, Tim Badgery-Parker, Deepak Bhonagiri, Craig Nelson, Wei Chua, Bradley Christian, Kate Churruca, Jeffrey Braithwaite

**Affiliations:** 1 Macquarie University, Sydney, Australia; 2 UNSW Sydney, Sydney, Australia; 3 New South Wales Department of Health, North Sydney, Australia; 4 Monash University, Melbourne, Australia; 5 University of Leeds, Leeds, United Kingdom; 6 Townsville Hospital, Townsville, Australia; 7 South Western Sydney Local Health District, Sydney, Australia; 8 Western Health, Melbourne, Australia; 9 The University of Sydney, Sydney, Australia

**Keywords:** Virtual care, Telehealth, Virtual care integration, Study protocol, Healthcare outcomes, Healthcare access, Healthcare equity, Digital health.

## Abstract

**Introduction:**

Virtual models of care comprise consultation by telephone, video-conferencing and remote-monitoring of health conditions, which are supported by digital patient information and wearable devices. Integration of virtual and in person care across health systems is a priority to create and sustain healthy nations by improving access to services, along with healthcare experiences, efficiency, and outcomes. Our collaborative project between health services, agencies, consumers, and clinicians across Australia seeks to provide the required evidence and solutions to optimise the integration of virtual care in hospital outpatient settings

**Methods and analysis:**

Our five-year project contains three embedded sub-studies that use a multi-method approach. Firstly, linked hospitalisation data will be used to produce fundamental new knowledge of patterns of virtual outpatient care use and the associated health service outcomes, including for priority populations. The second sub-study will use realist evaluation to determine the context, circumstances, and populations in which virtual care is used successfully, and economic impact of virtual hospital care for communities. We will then test the effectiveness of a co-designed Specialised Change Methodology for improving workforce change readiness and capability for integrating virtual models of care compared to current practice within health redevelopment settings. Statistical and qualitative analytic techniques will be applied with the resulting data to address the project aims.

**Ethics and dissemination:**

Ethics approval has been obtained (Study 1: HREC/97793/DOH-2023-383794; Study 2: 520231303852269; Study 3 520231586954286). Research dissemination will be channelled through established communities of practice in Australian states with whom the project is connected to reach networks of clinicians, consumers and health managers. Further targeted outputs will be devised in collaboration with the projects consumer, clinician and health system partners to guide the implementation and use of virtual modalities in outpatient hospital care, with equity as a central consideration.

**Supplementary Information:**

The online version contains supplementary material available at 10.1186/s12913-025-13129-2.

## Strengths and limitations of this study

- Partnership with clinicians, consumers and health agencies nationally will drive the project aims to be relevant to health system stakeholders.
- Using multiple hospital administrative datasets and data linkage methods enables this project to explore outcomes associated with virtual care across multiple states and services nationally but cannot provide causal evidence.
- Realist evaluation will enable the identification of the factors that influence virtual care experiences and outcomes for different communities.
- The analysis of hospital administrative data focuses on virtual care in outpatient settings only. Although findings may not be directly transferable to other virtual care provision in the Australian health system, or to other health systems learning from this research will have relevance internationally as many health services across the globe move to virtual delivery for some care processes.

## Introduction

Virtual models of healthcare delivery have been employed throughout Australia for many years to provide care to regional and remote locations [1]. Rapid scale up and adoption of virtual care internationally, and in metropolitan areas of Australia in response to the COVID-19 pandemic has utilised evidence from Australia’s long history of telehealth. As we transition into the post-COVID era, health systems recognise the opportunity for notable gains in efficiency and to redress inequities by appropriately employing virtual and hybrid models of service delivery with the required support [2]. Evidence of the outcomes and costs (both human and financial) associated with virtual care is critical in informing decisions about the which digitally enabled services should be sustained and how these can be integrated into routine care processes [3].

In hospital outpatient services, the use of virtual care has been associated with improved patient and clinician experiences, reduced costs and enhanced clinical outcomes for people with chronic and long-term conditions [4–6]. Enhanced patient experiences have been reported as a result of reduced financial burden associated with less travel required to receive care, childcare and other respite care costs [7–9]. Patients and carers also report opportunities for family members who cannot be physically present due to geographic distance, work or other commitments to attend and provide support when aspects of care are provided virtually [10]. For healthcare professionals, the reported benefits of digital solutions include the ability to access multidisciplinary teams and expertise at distance, along with reductions in travel time to attend patient visits. There is great potential for improved provision of care for people who have high healthcare utilisation, live at distance from major hospitals, and/or have multiple caring commitments. Yet evaluations of virtual care delivery to date indicate that benefits associated with using virtual care are not universal; they vary by population groups, personal circumstances, and care model [11–13]. Issues of digital exclusion, health literacy and socio-economic factors are identified barriers to successful realisation of the gains of virtual care for many individuals and families [14].

Desire for widespread use of virtual care has created an imperative to deliver robust evidence of the optimal use of virtual care and its implementation in hybrid models of service delivery. Health systems must also identify solutions to known implementation barriers that influence the success of virtual care integration with the existing services [15–17]. Evolving systems, processes and technologies have led to a piecemeal implementation approach between healthcare districts and services, with a lack of evidence-based approaches [18]. Historically, a lack of prospective planning for virtual care in health facility development or redevelopment continues to be a major contributing factor; integrating new technology into ageing infrastructure constrains the realisation of the virtual care benefits of improved patient experiences, health outcomes and reduced costs. In our analysis of statewide virtual care services, including remote monitoring, virtual hospitals, hospital-in-the-home and tele- or video-conferencing across > 20 specialities, a central barrier to the optimal use of virtual models was the limitations in the infrastructure design of healthcare facilities, along with ongoing challenges of limited equipment, connectivity and electronic health record integration [11]. These facility design barriers included lack of sufficient private space for telephone or video-consultations, and reception/waiting areas not designed to integrate the management of both face-to-face and virtual appointments. As the uptake and integration of virtual care evolves, uncertainty about the number of patients that will use virtual care and for what purpose, remains a challenge in updating facilities for virtual care use [19].

In a new era of virtual care at-scale, current fragmented approaches limit the extent to which system-wide cost savings and improvements in healthcare access and patient outcomes may be realised. Illuminated in the *NSW Health 20-Year Health Infrastructure Strategy* is the need for capital assets that are fit-for-purpose for future health [20]. The *Australasian Health Facility Guidelines* provide a common set of best-practice principles for health facility planners regarding the physical environment [21]. While these guidelines provide guidance on elements of virtual care planning and design, they are not designed to address all technological innovations, and the extent of their application varies between jurisdictions. Internationally, transformative infrastructure innovation is now critical from capital works investment and facility planning to accommodate integrated virtual care provision [18, 20].

Virtual healthcare delivery does not mimic in-person care; it requires changes to facility design but also in staff and consumer behaviours [22]. To deliver care using virtual or hybrid virtual and in person approaches, healthcare staff must adapt their practice to interact with a range of technologies to deliver and share health information, and to interact with patients, families and other healthcare workers [13, 23]. Engaging in virtual care therefore requires new individual and collective behaviours that may be a substantial departure from current practice [11–13]. Behavioural change can be supported by theory-based implementation science and evaluation techniques [24, 25]. Evidence from our systematic review of the management of healthcare change in the context of virtual care highlights that combining strategic change frameworks with implementation science methods provides a useful approach for supporting behavioural change amongst healthcare staff, but lacks application in shifting into greater use of virtual care [26, 27].

Population-based, robust evidence of the patterns of virtual care use in outpatient services and their associated impacts on health service delivery outcomes are needed to inform the integration of virtual care in health systems. Evidence of the differential contexts and mechanisms that contribute to virtual care experiences and outcomes is critical to understand how to best support virtual care implementation, along with processes to facilitate staff behavioural change. The Smarter Hospitals Project seeks to provide evidence to address these gaps through a five-year, national research program.

## Methods and analysis

### Study design

#### Study 1

Will be a retrospective cohort study of outpatient virtual care use in Victoria and Queensland among four patient cohorts with chronic or long-term conditions: people accessing cancer, renal, rehabilitation or mental health services. The study aims to describe the patterns of outpatient virtual care use among these patient cohorts over time: before, during and after the acute Covid-19 pandemic period, and the associated health service outcomes. The study will also investigate whether virtual care use and service-related outcomes vary between population groups including culturally and linguistically diverse communities, people with disabilities, by age, geographic location or disease type/stage. 

#### Study 2

**Fig. 1:**
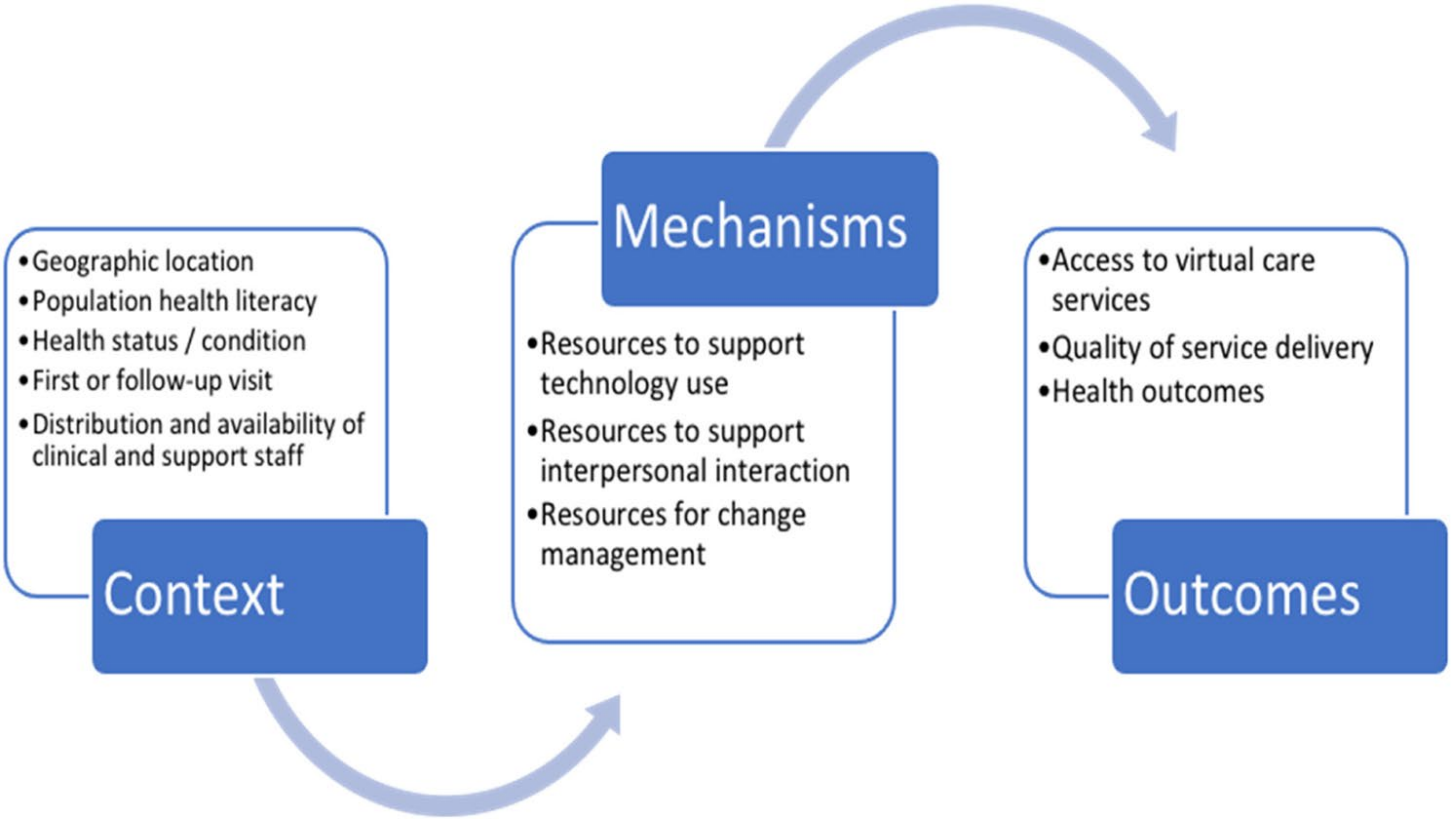
Contextual factors and mechanisms that are identified as driving outcomes in virtual care

Realist evaluation will be used to determine whether, how, when, where and why virtual care provision is effective. A realist evaluation explores interventions using the context-mechanism-outcome model; proposing that a given outcome is achieved because of underlying mechanism(s) that are operationalised in specific contexts [28]. Our preliminary work indicates that virtual care models can improve patient health and service delivery outcomes when certain mechanisms are apparent in certain contexts (Fig. 1). This information has been used to develop our initial theory about how virtual models might work to produce improved patient and service outcomes [11]. We will evaluate the virtual care models used by our health service partners to determine the conditions and circumstances that support their use, and populations for whom they work optimally [29, 30]. Using realist evaluation, which we have successfully employed previously for complex program evaluations [29, 31], we will test and refine our initial theory. Further, we will examine the economic impact of virtual care by using a modified Social Accounting Matrix framework to measure the incremental direct and indirect costs and benefits of implementing the virtual care models versus usual care. The economic analysis will include an activity-based costing study to identify the costs of developing and implementing virtual care models, exploring changes in health service costs, and evaluate the health and non-health out-of-pocket costs for consumers and their families.

#### Study 3

A controlled pre- and post-intervention study will be used to evaluate the use of a Specialised Change Methodology in improving workforce change readiness (comprising two components: change self-efficacy and commitment to change) in the context of hospital redevelopments that comprise substantial integration of virtual care. A mixed-methods survey and interview study will measure differences in workforce change readiness between cohorts of change managers, comparing change readiness among those using the Specialised Change Methodology and usual practice.

### Setting and data sources

#### Study 1

Outpatient hospital services providing cancer, renal, rehabilitation and mental health care in two Australian states: Victoria (VIC) and Queensland (QLD). We may seek to extend the research to include New South Wales (NSW) data in year two of the project subject to ethics and governance approvals. Data sources from Victoria and Queensland are: Victorian non admitted hospital services dataset, Victorian clinical public mental health services dataset, Victorian admitted episode dataset, Victorian emergency minimum dataset, Victorian cancer registry, Victorian registry of births, deaths and marriages, national cause of death unit record file for Victorians, Queensland health non-admitted patient data collection, Queensland consumer integrated mental health and addiction dataset, Queensland hospital admitted patient data collection, Queensland emergency data collection, Queensland cancer register and Queensland death registration and coded cause of deaths data.

#### Study 2

National study of people accessing outpatient cancer, renal, rehabilitation and mental health care in Australia.

#### Study 3

Two major hospital redevelopment projects in the Australian state of New South Wales.

### Study sample

#### Study 1

The data of patients aged ≥ 18 who attended outpatient clinics for the following priority chronic or long-terms conditions: (i) renal care; (ii) cancer; (iii) mental health; and (iv) rehabilitation in public hospitals over three-time periods will be eligible for inclusion. The time periods represent pre-, during and post- the acute Covid-19 pandemic period: 1 January 2017 and 31 December 2018 (T1), between 1 January 2020 and 31 December 2021 (T2) and between 1 January and 31 December 2023 (T3). These four outpatient services were identified by our healthcare partners as those with the greatest potential for improving access, quality and efficiency using virtual care because of the nature of these chronic and long term conditions, service demand and local burden of disease [32]. A sample of 395 patients is sufficient to identify a small effect size with 80% power (α = 0.05). Hence, the full dataset will provide adequate power to explore interactions and subgroup analyses.

#### Study 2

Approximately 60 consumers who have used virtual outpatient hospital services at a range of time points from 2017 onwards for one or more of the four conditions will be recruited. Sampling will seek to ensure representation from consumers with diverse cultural, ethnic, socioeconomic backgrounds, age groups, gender and sexuality, and ability, from a range of geographic regions and Australian states.

#### Study 3

Eligible staff are those who are directly involved in delivering the redevelopment and include: (1) hospital redevelopment staff (*n* = 20 per site), (2) NSW Health Infrastructure (HI; agency responsible for state-wide capital works) Project User Group (PUG) leads who chair stakeholder meetings called PUGs for HI throughout the design and redevelopment process to enable shared agreement between developers, architects, staff and consumers about the hospital design (*n* = 20 per site), and (3) staff who will be providing care using virtual and hybrid models at the newly redeveloped hospitals once completed (*n* ≥ 1000) staff.

### Recruitment

#### Study 1

Patient cohorts (cancer, renal, mental health and rehabilitation) were identified from the non-admitted patient data collection and the mental health ambulatory data collection for each state based on the patient service establishment unit type and/or non-admitted service classification. Cohort data were linked to emergency department (ED) presentation, hospital admission, and mortality data in each state for the three time periods by the Centre for Victorian Data Linkage (CVDL) and the Statistical Services Branch (SSB) Queensland. Linked data have now been received, and analysis is currently underway.

#### Study 2

Recruitment will occur through a multi-modal strategy that includes use of social media, healthcare and professional networks and newsletters to provide study information to people accessing the relevant virtual care services. Targeted recruitment of people who live regionally, rurally, who are from culturally and linguistically diverse backgrounds and/or have a disability will occur through a range of organisations including Language Other Than English Agency (LOTE), the Agency for Clinical Innovation (ACI) Intellectual Disability Network and a range of consumer groups. In each instance, a study flyer will be distributed via mailing lists and on social media groups, using plain English or translation as required. Recruitment for Study 2 is expected to commence shortly.

#### Study 3

Recruitment will occur via an email invitation distributed to hospital redevelopment staff, clinicians and change managers by NSW Health Infrastructure, which will contain links to the anonymous survey and unique identifiers to link data provided at each timepoint. Recruitment for Study 3 is expected to commence shortly.

### Data collection

#### Study 1 observational data

Retrospective, linked outpatient data will be securely transmitted by CVDL, and SSB to a secure research environment known as the Secured Unified Research Environment (SURE) or the Victorian Data Access Linkage Trust (VALT). The data will be exclusively accessible to authorised research team members.

#### Study 2 interviews

Our research team will use preliminary discussions with potential participants during the recruitment process to determine the support needs and preferences of individuals ahead of undertaking consent processes and scheduling interviews. Once preferences and needs are identified and addressed, interviews will be conducted using video-conferencing software or in person based on the participant’s preferences and requirements. Interview will be conducted by the research team, supported by accredited translators and support persons as required. Participants and their supporters will be reimbursed and remunerated for involvement as per the Health Consumers NSW payment guidelines. For online interviews, video-conferencing software will be used for transcription. For in person interviews, audio-recorders will be used, and the data will be sent to a professional transcription service (Pacific Transcription) or using transcription software.

#### Study 3 surveys

##### Intervention group

**Fig. 2:**
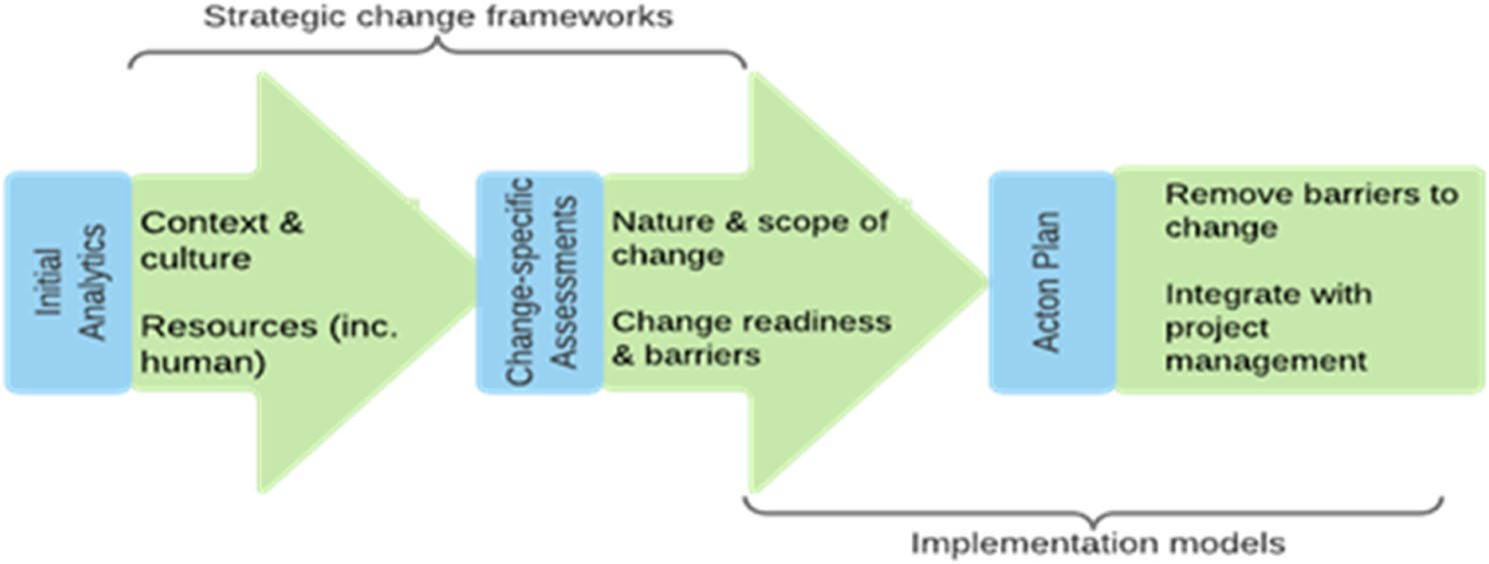
Three stages of AM specialised change methodology

Redevelopment staff and PUG leads at Hospital Redevelopment Site A (*n* = 40) will receive training in the *Specialised Change Methodology*. The *AM Specialised Change Methodology* is a theory-based methodology that aims to improve change readiness in infrastructure projects. The methodology was co-produced by the investigator team and HI, and integrates models from strategic change management, implementation science and project management in three stages (Fig. 2). It will be used by redevelopment staff and PUG leads at one redevelopment site over 12-months to increase change readiness for integrating virtual models.

##### Control group

A matched sample of redevelopment staff and PUG leads (*n* = 40) at Hospital Redevelopment Site B, in which no formalised change methodology will be implemented, will provide comparator outcome data over a 12-month period.

##### Outcome measures

Surveys and semi-structured interviews will be used to gather pre- and post-intervention outcome data from participating staff.

i. *Online surveys*: Organisational readiness for integrating virtual care models will be measured using validated surveys previously deployed to capture individual and collective change readiness [33]. These comprise: (i) a 4-item individual change readiness scale, (ii) a 4-item collective change readiness scale; (iii) a 4-item change self-efficacy scale; and (iv) a 3-item affective commitment to change scale [33–35]. Surveys will be administered at three time-points at both intervention and control sites: during redevelopment planning (T1), during redevelopment design (T2), and at commencement of service provision (T3). For 80% power to detect a small to medium effect (α = 0.05), we will aim to survey 80 healthcare staff at each time point.
ii. *Semi-structured telephone interviews* (at pre- and post-intervention) will be undertaken with the 40-redevelopment staff and 40 PUG leads per site. Thematic analysis of interview data will be undertaken to gather evidence of the acceptability, feasibility and perceived value of the *AM Specialised Change Methodology* and related training at the intervention site; to explore initial perceptions of change readiness; to establish the way in which change management processes occurred in each site and explain any variation between sites that may have influenced the findings [36]. An iterative process of data collection and analysis will guide sample size [37].

### Data analysis plan

#### Study 1 observational data

Descriptive analysis will be performed comparing the characteristics of study sample in the cohorts for three study periods. Chi-square tests of independence and ANOVA will be used to further describe the patient, neighbourhood and subsequent health service use outcomes. Generalised linear mixed modelling will be used to perform multi-level modelling to examine the association between type of care (in person, hybrid or entirely virtual) with health service outcomes (emergency department visits, inpatient admissions, length of stay, mortality), adjusting for covariates including an indicator variable for COVID 19, and considering clustering of patients within health service areas. Covariates included will be age, gender, country of birth, language spoken at home, economic status, comorbidity index, remoteness classification of the place of residence, area level socio economic disadvantage and any other relevant diagnosis related variables that could potentially influence the outcomes such as stage of cancer, type of mental health diagnosis. Two-way interactions between the variables will also be explored. To account for the COVID 19 related health service encounters, a sensitivity analysis will be undertaken excluding the service use due to COVID 19 (ICD 10 AM U07). Missing data will be handled using complete-case analysis. The extent and pattern of missingness will be examined, and sensitivity analyses will be conducted where appropriate to assess the robustness of findings. Adjusted relative risks and 95% confidence intervals will be reported.

#### Study 2 interview data

Retroductive analysis will be undertaken combining inductive and deductive approaches following Gilmore et al. [38] to reveal the possible causal factors that underpin the results generated by Study 1. Our analysis will provide insight into why different virtual models work in certain contexts/for certain groups to achieve improved health service outcomes. Outcomes of interest include subsequent planned and unplanned hospitalisation after outpatient virtual care, length of stay in these admissions, patient reported outcomes of care and patient reported experiences of care. Economic analysis will examine the economic impact of virtual care by using a modified Social Accounting Matrix (SAM) framework to measure the incremental direct and indirect costs and benefits of implementing the virtual care models versus usual care. All costs and benefits will be monetised and rebased to a reference year with a 5% discount rate. A sensitivity analysis will be conducted to examine variability, and a return on investment will be determined for implementing virtual models.

#### Study 3 survey data

Survey data will be analysed using descriptive statistics (e.g. mean, percent change) for each site and Mann-Whitney test to compare pre-post intervention. We will use multiple linear regression to estimate the effect of the *Specialised Change Methodology* adjusting for differences in individual factors such as age, experience, role (clinical or non-clinical), and time in role.

### Ensuring study quality

This program of work has been through an independent scientific peer review process by the National Health and Medical Research Council under the Partnership Projects Funding Scheme (Project number: 2015544). The scheme has competitively funded this research based on the scientific quality of the proposal and require progress reporting biannually. Throughout the project, study quality will be ensured by our project governance process which comprises primarily of an external stakeholder Project Steering Group (PSG) that includes consumers. The PSG meets twice a year to provide independent oversight of the project processes and progress against milestones. Consumer members specifically provide review and advice on consumer involvement activities and project processes to ensure that we retain a consumer-centric approach.

### Patient or public involvement

Consumer involvement has been central to all elements of the research process from the project inception to execution, with a range of mechanisms being used to ensure the research receives inputs from and is disseminated with diverse stakeholder groups and communities. The protocol has been co-authored with two consumer advisors representing cultural diversity, people with disability and long-term health conditions, who have informed the project approach (DDB and MS). Consumer input is embedded in the project governance through a Project Advisory Group has that includes two consumer members and provides oversight of the research approach throughout the project lifecycle. These members will also review any materials or processes of research proposed with patients and carers in detail throughout the research. During each study, community engagement with targeted populations is used to gain insight into the appropriate mechanisms to reach consumers and ensure that a diverse range of consumer perspectives are considered in the research. Attention to diverse representation is embedded in each study to ensure that participants are representative of the Australian population. Working in partnership with the project’s consumer advisors, a dissemination plan has been developed that embeds principles of accessible and inclusive research to ensure findings are shared and discussed with consumers broadly.

## Ethics and dissemination

Ethical considerations have been explored, identified and a risk mitigation plan created for each matter arising through the process of applying for ethical approval for the conduct of the study. Ethics approval has been obtained for all components of the research. The first study has ethical approval Queensland and Victorian Human Research Ethics Committees (HREC/97793/DOH-2023-383794), studies 2 and 3 have further been approved by Macquarie University Human Research Ethics Committee (Study 2: 520231303852269 Study 3: 520231586954286), which is a National Health and Medical Research Council (NHMRC) recognised ethics committee. During the study, data will be stored on the secure VALT or SURE systems (Study 1) or the OneDrive system (studies 2 and 3) of the leading institution with the primary investigator and retained in this secure location for at least seven years following the end of the project in accordance with the national ethical requirements.

Through the project development process, several key risks and mitigation strategies were identified and developed. Four strategies will ensure that research activities will be managed and coordinated effectively. Firstly, we have established approval from the research sites in each state to conduct this work to mitigate the risk of not being able to access the services and individuals within these. Secondly, to address risks of working nationally, we have local project team members in each state to ensure local oversight. Thirdly, we are cognisant of the complexities, associated risks and mitigation practices needed to work with a highly diverse consumer group. To address the risk of not being able to interact with the diverse target population of consumers effectively, we access relevant translation services, bilingual fieldworkers and have budgeted for the associated costs and complexity. Finally, annual meetings, monthly virtual meetings and the project reference group mitigates risk and enhances our ability to respond effectively.

The study findings will be disseminated at multiple events and through a range of formats to ensure that all stakeholder groups with interest in the project and its outcomes are able to access and interact with the findings. Specific mechanisms to enhance the successful implementation of virtual care will be disseminated, along with findings from the individual studies. In year 1, we will engage with established communities of practice around virtual care in three Australian states to translate knowledge as it emerges throughout the project into policy and practice change, and into business as usual with the partner organisations. COPs are a tool to facilitate the implementation of evidence-based practice across boundaries, but also to generate and manage evidence to support the adoption of best practice [39] Our approach to knowledge translation seeks to build clinician, facility management and research capacity to advance and sustain the adoption of virtual models using standardised and evidence-based approaches to improve service delivery, outcomes and system efficiency.

## Supplementary Information

Supplementary Material 1

Supplementary Material 2

## Data Availability

No datasets were generated or analysed during the current study.
