# Supplementary material for: Unlocking the promise of virtual care in hospitals: the Smarter Hospitals Project protocol
Source: BMC Health Serv Res. 2025 Aug 11;25:1058. doi: 10.1186/s12913-025-13129-2 (PMC12337380; doi:10.1186/s12913-025-13129-2)
Supplement: Supplementary file 1 — Supplementary Material 1 [file 12913_2025_13129_MOESM1_ESM.docx]

**Supplementary Table 1: STROBE Checklist**

This protocol includes both observational and qualitative studies. The observational study component has been prepared in accordance with the 'Strengthening the Reporting of Observational Studies in Epidemiology' (STROBE) guidelines. As this is a protocol paper, the STROBE checklist has been completed only where applicable, with the results and discussion sections left blank.

| **No** | **Item** | **Recommendation** | **Page No.** |
| --- | --- | --- | --- |
| 1 | Title and abstract | (a) Indicate the study’s design with a commonly used term in the  title or the abstract | 1-2 |
|  |  | (b) Provide in the abstract an informative and balanced summary  of what was done and what was found | 1-2 |
| **Introduction** | | | |
| 2 | Background/rationale | Explain the scientific background and rationale for the investigation being reported | 2-5 |
| 3 | Objectives | State specific objectives, including any prespecified hypotheses | 5 |
| **Methods** | | | |
| 4 | Study design | Present key elements of study design early in the paper | 5 |
| 5 | Setting | Describe the setting, locations, and relevant dates, including  periods of recruitment, exposure, follow-up, and data collection | 7 |
| 6 | Participants | (a) Give the eligibility criteria, and the sources and methods of  selection of participants | 7-8 |
| 7 | Variables | Clearly define all outcomes, exposures, predictors, potential  confounders, and effect modifiers. Give diagnostic criteria, if  applicable | 11 |
| 8 | Data sources/  measurement | For each variable of interest, give sources of data and details of  methods of assessment (measurement). Describe comparability  of assessment methods if there is more than one group | 11 |
| 9 | Bias | Describe any efforts to address potential sources of bias | 11 |
| 10 | Study size | Explain how the study size was arrived at | 7-8 |
| 11 | Quantitative variables | Explain how quantitative variables were handled in the analyses. If applicable,  describe which groupings were chosen and why | 11 |
| 12 | Statistical methods | (a) Describe all statistical methods, including those used to control for confounding | 11 |
|  |  | (b) Describe any methods used to examine subgroups and interactions | 11 |
|  |  | (c) Explain how missing data were addressed | 11 |
|  |  | (d) Cross-sectional study—If applicable, describe analytical methods taking account of  sampling strategy | 7, 11 |
|  |  | (e) Describe any sensitivity analyses | 11 |
| **Results** | | | |
| 13 | Participants | (a) Report numbers of individuals at each stage of study—eg numbers  potentially eligible, examined for eligibility, confirmed eligible, included in the  study, completing follow-up, and analysed | N/A |
|  |  | (b) Give reasons for non-participation at each stage | N/A |
|  |  | (c) Consider use of a flow diagram | N/A |
| 14 | Descriptive  data | (a) Give characteristics of study participants (eg demographic, clinical, social)  and information on exposures and potential confounders | N/A |
|  |  | (b) Indicate number of participants with missing data for each variable of  interest | N/A |
| 15 | Outcome data | Report numbers of outcome events or summary measures | N/A |
| 16 | Main results | (a) Give unadjusted estimates and, if applicable, confounder-adjusted  estimates and their precision (eg, 95% confidence interval). Make clear which  confounders were adjusted for and why they were included | N/A |
|  |  | (b) Report category boundaries when continuous variables were categorized | N/A |
|  |  | (c) If relevant, consider translating estimates of relative risk into absolute risk  for a meaningful time period | N/A |
| 17 | Other analyses | Report other analyses done—eg analyses of subgroups and interactions, and  sensitivity analyses | N/A |
| **Discussion** | | | |
| 18 | Key results | Summarise key results with reference to study objectives | N/A |
|  | Limitations | Discuss limitations of the study, taking into account sources of potential bias or imprecision. Discuss both direction and magnitude of any potential bias | N/A |
|  | Interpretation | Give a cautious overall interpretation of results considering objectives,  limitations, multiplicity of analyses, results from similar studies, and other  relevant evidence | N/A |
|  | Generalisability | Discuss the generalisability (external validity) of the study results | N/A |
| **Other Information** | | | |
| 22 | Funding | Give the source of funding and the role of the funders for the present study  and, if applicable, for the original study on which the present article is based | 15 |
