# Supplementary material for: Unlocking the promise of virtual care in hospitals: the Smarter Hospitals Project protocol
Source: BMC Health Serv Res. 2025 Aug 11;25:1058. doi: 10.1186/s12913-025-13129-2 (PMC12337380; doi:10.1186/s12913-025-13129-2)
Supplement: Supplementary file 2 — Supplementary Material 2 [file 12913_2025_13129_MOESM2_ESM.docx]

Strategic change frameworks

Implementation models

Figure 2 Three stages of Specialised Change Methodology
